# CUL5–ASB6 Complex Promotes p62/SQSTM1 Ubiquitination and Degradation to Regulate Cell Proliferation and Autophagy

**DOI:** 10.3389/fcell.2021.684885

**Published:** 2021-06-07

**Authors:** Liyan Gong, Kaihua Wang, Mengcheng Wang, Ronggui Hu, Huaguang Li, Daming Gao, Moubin Lin

**Affiliations:** ^1^Center for Clinical Research and Translational Medicine, Yangpu Hospital, Tongji University School of Medicine, Shanghai, China; ^2^Department of General Surgery, Yangpu Hospital, Tongji University School of Medicine, Shanghai, China; ^3^State Key Laboratory of Cell Biology, Shanghai Institute of Biochemistry and Cell Biology, Center for Excellence in Molecular Cell Science, Chinese Academy of Sciences, Shanghai, China; ^4^University of Chinese Academy of Sciences, Beijing, China; ^5^State Key Laboratory of Molecular Biology, Shanghai Institute of Biochemistry and Cell Biology, Center for Excellence in Molecular Cell Science, Chinese Academy of Sciences, Shanghai, China

**Keywords:** p62, ubiquitination, CUL5, ASB6, proliferation, autophagy

## Abstract

p62/SQSTM1 (sequestosome-1) is a key protein involved in multiple cellular bioprocesses including autophagy, nutrient sensing, cell growth, cell death, and survival. Therefore, it is implicated in human diseases such as obesity and cancer. Here, we show that the CUL5–ASB6 complex is a ubiquitin E3 ligase complex mediating p62 ubiquitination and degradation. Depletion of CUL5 or ASB6 induced p62 accumulation, and overexpression of ASB6 promoted ubiquitination and degradation of p62. Functionally, ASB6 overexpression can inhibit the proliferation of MEF and hepatocellular carcinoma cells by reducing p62 protein level, and impair the occurrence of autophagy. Overall, our study identified a new molecular mechanism regulating p62 stability, which may provide additional insights for understanding the delicate control of p62 and cell proliferation–autophagy control in physiological and pathological settings.

## Introduction

p62, encoded by *SQSTM1* gene, is the first discovered autophagic adaptor protein, which participates in many cellular processes, such as cell growth and proliferation, autophagy, malignant transformation, apoptosis, and inflammation (Layfield and Hocking, 2004; McManus and Roux, 2012; Moscat and Diaz-Meco, 2012). During the autophagy process, PB1 domain of p62 promotes the packaging of ubiquitinated substrates through oligomerization (Kraft et al., 2016), and LIR domain of p62 mediates its interaction with LC3, thus transporting the packaged substrates and participating in the formation of autophagosome (Park et al., 2014). It is reported that several kinases including CK2/TBK1 and ULK1 phosphorylate p62 at Ser403 and Ser407 within the UBA region of p62, promoting p62 ubiquitination and the subsequent autophagy degradation (Matsumoto et al., 2011; Pilli et al., 2012; Ro et al., 2014; Lim et al., 2015). In addition to autophagy regulation, p62 interacts with receptor interacting protein (RIP) and connects with aPKCs to activate tumor necrosis factor α (TNFα)-induced NF-κB signaling pathway (Sanz et al., 1999). On the other hand, p62 recruits TRAF6, the inflammation signaling molecule and E3 ubiquitin ligase, and promotes TRAF6-dependent ubiquitination and activation of mTORC1 under amino acid-rich conditions (Jadhav et al., 2008; Linares et al., 2013). Moreover, the interaction between p62 and Keap1 can destroy Keap1-mediated ubiquitination of Nrf2, leading to Nrf2 activation (Jain et al., 2010). Reciprocally, Nrf2 can enhance the expression of p62 at the transcription level by directly binding to the promoter region of *p62/SQSTM1* gene, forming a positive feedback loop (Liu et al., 2007). Therefore, p62 acts as a multifunctional signaling hub involved in nutrition sensing (*via* mTORC1), inflammation and apoptosis (*via* NF-κB), and antioxidant response and selective autophagy pathways (*via* Keap1-Nrf2). Since alterations of all these important pathways are associated with human diseases such as cancer, no surprise p62 has been shown playing a role in tumorigenesis. More and more evidences indicate abnormal expression of p62 in various cancers, including liver (Inami et al., 2011), lung (Inoue et al., 2012), breast (Rolland et al., 2007), kidney (Li et al., 2013), colorectal (Ren et al., 2014), ovarian (Yan et al., 2019), and prostate cancers (Kitamura et al., 2006). For example, p62 accumulation can destabilize the genome and promote tumor development; p62 can mediate tumor-induced fat reprogramming in adipocytes and has a potential impact on obesity-promoted cancer (Komatsu, 2011; Huang et al., 2018). Importantly, increased p62 expression in cancer cells is regarded a consequence of defective autophagy, which promotes tumorigenesis (Mathew et al., 2009). Recent results from liver cancer mouse models suggest that high p62 expression exerts its oncogenic activity *via* Nrf2, mTORC1, and c-Myc activation, and hepatocyte-specific deletion of p62 impairs hepatocellular carcinoma (HCC) formation (Umemura et al., 2016). Consistently, elevated p62 levels are often observed in HCC and liver diseases with increased risk of malignant transformation (Aigelsreiter et al., 2017; Sanchez-Martin et al., 2019). Therefore, the de-regulated p62 may be a potential therapeutic target for HCC.

Ubiquitination is a major post-translational modification regulating protein properties including stability, interaction spectrum, localization, and so on. Protein ubiquitination is typically catalyzed by ubiquitin-activating enzymes (E1s), ubiquitin-conjugating enzymes (E2s), and ubiquitin ligase enzymes (E3s) (Hershko and Ciechanover, 1998; Schulman and Harper, 2009; Wenzel et al., 2011). E3 ubiquitin ligases are the most heterogeneous class of enzymes in the ubiquitination pathway, since they control the substrate specificity (Morreale and Walden, 2016). Several E3 ubiquitin ligases have been identified to modulate the expression or functions of p62. Keap1-Cullin3 ubiquitylates p62 at K420, leading to diminished p62 sequestration and degradation activity during autophagy (Lee et al., 2017). TRIM21 and NEDD4 were reported to mediate ubiquitylation of p62 at K7, leading to suppressed protein sequestration and induced inclusion body autophagy (Pan et al., 2016; Lin et al., 2017). The E3 ligase RNF26 ubiquitylates p62 within the UBA domain to facilitate TOLLIP interaction and vesicular cargo sorting (Jongsma et al., 2016), while RNF166 ubiquitylates p62 to modulate the role of p62 in xenophagic targeting of bacteria (Heath et al., 2016). In addition to the E3 ligases that modulate p62 activity, two E3 ligases have been reported to regulate p62 stability *via* proteasomal degradation. The E3 ubiquitin ligase Parkin directly interacts with and ubiquitinates p62 to promote proteasomal degradation of p62, and dysregulation of Parkin/p62 axis could account for the selective vulnerability during pathogenesis of PD (Song et al., 2016). Another recent study has shown that X-linked inhibitor of apoptosis protein (XIAP) functioned as a ubiquitination E3 ligase toward p62 and suppressed p62 expression through ubiquitin-proteasomal degradation and therefore promoted breast cancer progression (Huang et al., 2019). Therefore, p62 is ubiquitinated in various physiological settings. In the current study, we found that a functional Cullin-Ring E3 ligase (CRL) complex composed of Cullin5 (CUL5), Elongin B (EloB), Elongin C (EloC), and substrate recognition adaptor ASB6 interacts with p62 and mediates its ubiquitination-dependent degradation. Our experimental evidences indicate that ASB6 overexpression inhibits the proliferation of HCC cells and impairs autophagy by reducing the p62 protein levels. Therefore, our study has not only characterized a new functional CRL5–ASB6 E3 complex, but also identified p62 as the first degradation substrate of it, which may provide new insight for cell proliferation and autophagy regulation.

## Materials and Methods

### Reagents, Antibodies, and Plasmid Constructs

DMSO and cycloheximide (CHX) were purchased from Sigma. MG132 and MLN4924 were purchased from Selleck Chemicals. Bafilomycin A1 (Baf A1) was purchased from Sigma. DMEM (Dulbecco’s Modified Eagle Medium), DMEM/F-12 (Dulbecco’s Modified Eagle Medium/Nutrient Mixture F-12), FBS (Fetal Bovine Serum), Penicillin–Streptomycin, and puromycin were purchased from Invitrogen (Thermo Fisher Scientific). Transfection reagent polyethylenimine was purchased from Sigma. Lipofectamine 3000 was purchased from Thermo Fisher Scientific, and siRNA transfection reagent X-tremeGENE was purchased from Roche.

The following antibodies were used for Western blot: p62/SQSTM1 (catalog A11250) was from ABclonal; ASB6 (catalog 21449-1-AP), HA (catalog 51064-2-AP), Myc (catalog 16286-1-AP), and GFP (catalog 66002-1) were from Proteintech; Tubulin (catalog SC23948), CUL5 (catalog SC-373822), and HA (catalog SC-7392) were from Santa Cruz Biotechnology; FLAG (catalog F3165), FLAG (F7425), Vinculin (catalog V4505), and His (catalog H1029) were from Sigma; p27/kip1 (catalog 610241) was from BD.

Human p62 (including p62 and p62S), CUL5, ASB6, EloB, or EloC were PCR amplified and inserted into the pcDNA3.1, pCMV-FLAG, or pLEX-MCS-FLAG vectors. shRNA vectors were generated by inserting synthesized oligos into pLKO.1 vector. The shRNA target sequences for CUL5 were 5′-GCCATCAAGATGATACGGCTT-3′, 5′-GCTAGAATGTTTCAGGACATA-3′, and 5′-GAGGAACATA TCATTAGTGC-3′. The shRNA target sequences for EloB were 5′-CCAACTCTTGGATGATGGCAA-3′ and 5′-CGAACT GAAGCGCATCGTCGA-3′. The shRNA target sequences for EloC were 5′-CGAAACCAATGAGGTCAATTT-3′ and 5′-CGTACAAGGTTCGCTACACTA-3′. The shRNA target sequences for ASB6 were 5′-GCAGATCCACAATACTGA GAA-3′, 5′-CCCGAAAACTTCGATATCCAC-3′, 5′-AGGAG AGCCGAATCCTTGTTC-3′, and 5′-CACAGTGTTCACCT GCATCAT-3′. The shRNA target sequence for p62 was 5′-CCTCTGGGCATTGAAGTTGAT-3′.

### Cell Culture and Transfection

HeLa, HEK293T, and HepG2 cells were cultured in DMEM at 37°C/5% CO_2_, while SNU739, SNU182, and Huh1 cells were cultured in DMEM/F-12 at 37°C/5% CO_2_. All culture media were added with 10% FBS and 1% penicillin/streptomycin before use. Transfection experiments were performed when the cells were about 60–80% confluent. According to different cell types, we choose different transfection reagents and methods. HEK293T and HeLa cells were transfected with polyethylenimine and Lipofectamine 3000 reagents, respectively. siRNAs were transfected into cells with X-tremeGENE siRNA Transfection Reagent at 50 nM final concentration according to the manufacturer’s protocol. The siRNA sequences targeting ASB6 were as follows: 5′-CAGAUCCACAAUACUGAGA-3′ and 5′-C CGAAAACUUCGAUAUCCA-3′.

### Western Blotting and Immunoprecipitation (IP)

Cells were harvested in EBC lysis buffer (50 mM Tris-HCl, pH 8.0, 120 mM NaCl, and 0.5% Nonidet P-40) supplemented with protease inhibitors (Selleck Chemicals) and phosphatase inhibitors (Selleck Chemicals) to generate cell lysates. Protein concentration of cell lysates was measured using Bio-Rad protein assay kit in a spectrophotometer (Thermo Scientific). Equal amounts of protein were resolved by electrophoresis on SDS-PAGE gels and transferred onto a PVDF membrane. After incubation in blocking buffer [50 mM Tris-buffered saline (pH 7.4) containing 5% non-fat dry milk and 0.1% Tween-20], the membranes were probed with the primary antibodies, followed by incubation with HRP-conjugated rabbit or mouse secondary antibodies. For immunoprecipitation, cell lysates were incubated with anti-FLAG M2 agarose beads or anti-HA agarose beads for 3 h. Beads were then washed five times with NETN buffer (20 mM Tris-HCl, pH 8.0, 100 mM NaCl, 1 mM EDTA, and 0.5% NP-40). After washing, the precipitated samples were resolved on SDS-PAGE and immunoblotted with appropriate antibodies.

### Lentiviral Production and Infection

Lentiviral packaging and infection were done as previously described (Du et al., 2015). Briefly, HEK293T cells were co-transfected with pLKO.1 or pLEX constructs and the packaging plasmids psPAX2 and pMD2.G. All media were removed after 5 h and replaced with fresh DMEM plus 10% FBS. Virus containing medium were collected and filtered with a 0.45-μm membrane (Merck Millipore) 48 h after replacement with fresh media. Polybrene (10 μg/ml) was added into the virus-containing medium to infect the corresponding cells, and infected cells were selected in puromycin for 48 h before harvest or following experiments.

### Cell Proliferation Assay

For cell proliferation assays, 500 cells were seeded in 96-well plates (Nest), and the viability of the cells was measured at various time points. Ten microliters of CCK8 (Meilunbio) reagent was added to each well, and the cells were incubated at 37°C for 2 h. Next, absorbance was measured in single-wavelength mode (450-nm) using a BioTek Eon Multi-Mode Microplate Readers.

### Colony Formation Assay

For cell colony formation assays, 500 cells were seeded in each well of six-well plates, and cultured for 10 days until visible colonies formed. Colonies were then washed with PBS, fixed, and stained with 0.1% crystal violet for 20 min. After staining, the plates were washed with distilled water and air-dried. Visible colonies were counted.

### Immunofluorescence Analysis

Cells were grown on glass coverslips for treatment as indicated and then fixed with 4% paraformaldehyde in PBS for 15 min at room temperature and permeabilized with 0.5% Triton X-100 in PBS for 5 min. Samples were rinsed three times with PBS (5 min each time). Coverslips were then blocked for 60 min with 5% BSA. After washing three times with PBS (10 min each time), nuclei were counterstained with 4,6-diamidino-2-phenylindole (DAPI) for 10 min. Coverslips were rinsed twice (3 min for each wash) with PBS and mounted onto slides using ProLong Gold Antifade reagent (Invitrogen). All images were obtained with the Leica TCS SP8 fluorescence microscope.

### Statistical Analysis

Each experiment was repeated at least three times, and results were presented as mean ± standard error of the mean. The statistical significance of differences was assessed by the Student’s unpaired *t*-test (^∗^0.01 < *P* < 0.05, ^∗∗^0.001 < *P* < 0.01, and ^∗∗∗^*P* < 0.001). Statistical analysis was performed using GraphPad Prism.

## Results

### p62 Is Modulated by CRL5 E3 Ligase Complex

**FIGURE 1:**
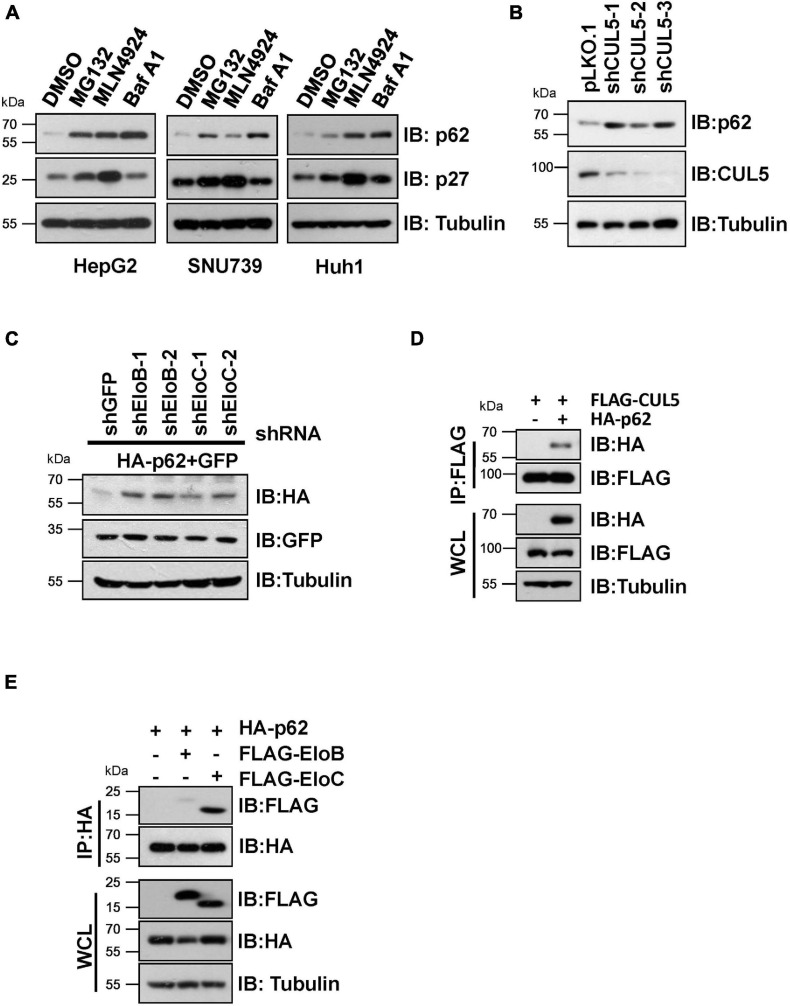
CRL5 interacts with p62 and regulates its expression. **(A)** HepG2, SNU739, and Huh1 cells were treated with 10 μM MG132, 1 μM MLN4924, or 200 nM Baf A1 for 10 h, and the whole-cell lysates (WCL) were generated for immunoblotting (IB) analysis. p27 served as a positive control responding to CRL E3 blocking, and Tubulin served as a loading control. **(B)** Huh1 cells infected with indicated shRNAs were subjected to IB analysis. Tubulin served as a loading control. **(C)** IB analyses of WCL from HeLa cells transfected with plasmids encoding HA-p62, shRNAs targeting EloB/C, and GFP (as an internal transfection control). Tubulin served as a loading control. **(D,E)** HEK293T cells transfected with the indicated plasmids were treated with 10 μM MG132 for 10 h before Co-IP and IB analysis.

We initially found that proteasome inhibitor MG132, the NEDD8-activating enzyme inhibitor MLN4924 (often used to suppress CRL E3 ligase activity) (Soucy et al., 2009), and the autophagy inhibitor bafilomycin A1 (Baf A1) caused an obvious elevation of endogenous p62 protein levels in HepG2, SNU739, and Huh1 cells (Figure 1A), indicating that p62 is an unstable protein that is likely governed by CRL E3 ligase complexes. Moreover, knockdown of CUL5 could upregulate endogenous p62 protein level and co-transfection of shRNAs against EloB/EloC in HeLa cells can dramatically upregulate the expression of ectopically expressed HA-p62 (Figures 1B,C). Then, we further confirmed the interaction between p62 and the CUL5–EloB/EloC complex by transfection/co-immunoprecipitation (IP) experiments (Figures 1D,E). Notably, the interaction between p62 and EloC was stronger than the p62–EloB interaction. These results indicated that p62 is a potential CRL5 ubiquitination substrate, since the substrate recognition subunit of CRL5 directly interacts with the linker protein EloC *via* the SOCS-box region and indirectly interacts with EloB *via* N-terminus of CUL5 protein.

### ASB6 Is the SOCS-Box Protein That Interacts With p62

**FIGURE 2:**
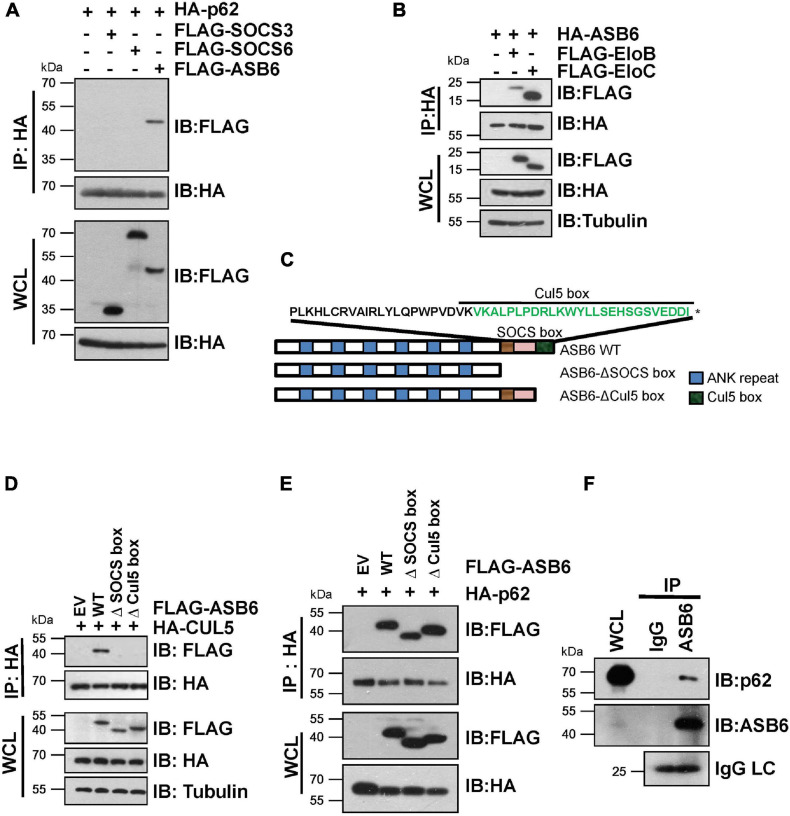
ASB6 interacts with p62 and it is part of CRL5 E3 ligase complex. **(A,B)** HEK293T cells transfected with the indicated plasmids were treated with 10 μM MG132 for 10 h before harvest for Co-IP and IB analysis. **(C)** Schematic illustration of human ASB6 protein domain composition. **(D,E)** HEK293T cells were co-transfected with ASB6 wild type and mutants (Δ-SOCS box, Δ-Cul5-box) and HA-CUL5 **(D)** or HA-p62 **(E)** constructs. Cells were treated with 10 μM MG132 for 10 h before harvest for Co-IP and IB analysis. **(F)** Huh1 cells were treated with 10 μM MG132 for 10 h before harvest to make whole-cell lysates. ASB6 antibody was used to perform endogenous Co-IP experiments with Rabbit IgG as negative control.

In order to identify the substrate recognition subunit that specifically mediates CRL5-dependent p62 regulation, we performed shRNA-based screening and three SOCS-box proteins were identified, including SOCS3, SOCS6, and ASB6. By co-immunoprecipitation (Co-IP) experiment, a strong interaction was observed between p62 and ASB6, but not SOCS3 or SOCS6 (Figure 2A). Meanwhile, HA-ASB6 interacts with both FLAG-EloB and EloC, and the binding affinity of HA-ASB6 to EloC is significantly stronger than to EloB (Figure 2B), a pattern very similar to the interaction of p62 with EloB/EloC (Figure 1E). According to the domain composition of ASB6 (Figure 2C), we generated internal deletion FLAG-ASB6 constructs and co-transfected them with HA-CUL5 or HA-p62 in HEK293T cells to examine the interaction between ASB6 with CUL5 and p62. The Co-IP results suggested that removing SOCS box or Cul5-box domain abolished the ASB6–CUL5 interaction, but did not impair the interaction between ASB6 and p62 (Figures 2D,E). In order to test whether ASB6 is indeed an endogenous interacting protein of p62, we next performed Co-IP experiment with lysate generated from human liver cancer cell line Huh1, in which the tumor-promoting effect of p62 has been validated. As indicated, indeed ASB6 binds to p62 at endogenous level (Figure 2F). These results suggest that ASB6 may be the key SOCS box protein in the CRL5 E3 complex that binds and regulates p62.

### ASB6 Promotes Ubiquitination and Degradation of p62

**FIGURE 3:**
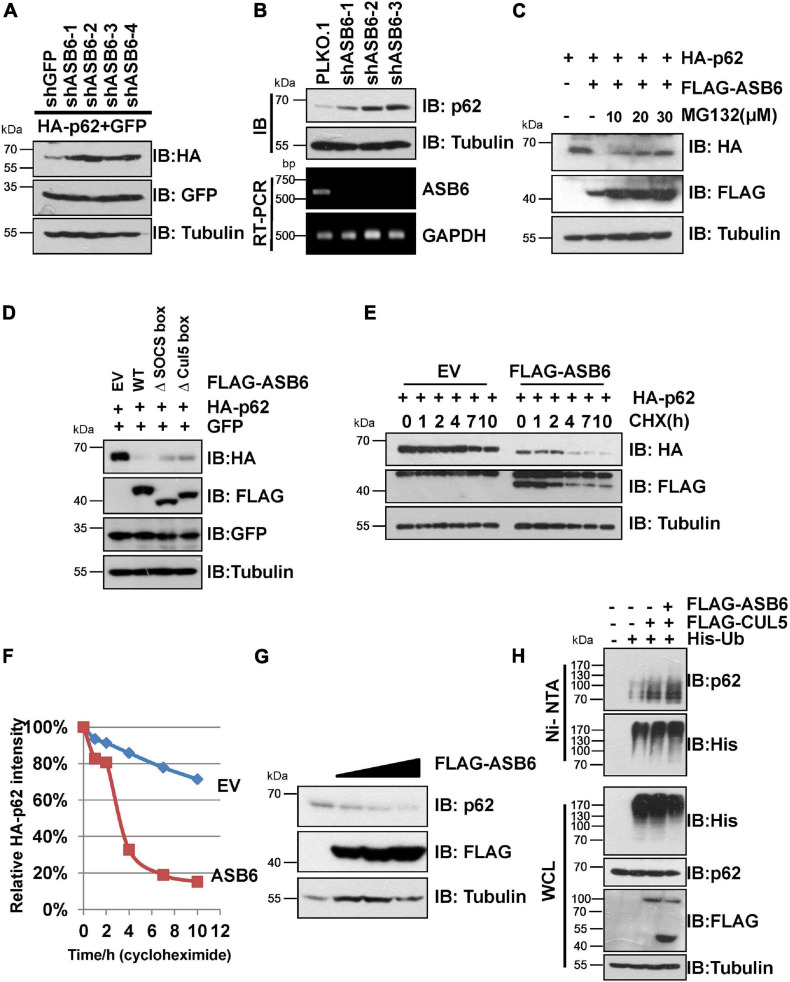
CUL5–ASB6 complex promotes ubiquitination and degradation of p62. **(A)** IB analyses of WCL from HEK293T cells co-transfected with plasmids encoding HA-p62, ASB6 shRNAs, and GFP (as an internal transfection control). Tubulin served as a loading control. **(B)** HeLa cells infected with indicated shRNAs were harvested for IB analysis. Tubulin served as a loading control. The knockdown efficiency of ASB6 was examined by RT-PCR. *GAPDH* gene was detected as the internal control. **(C)** HEK293T cells were co-transfected with plasmids encoding HA-p62 and FLAG-ASB6 and were treated with different concentrations of MG132 (10, 20, and 30 μM) for 10 h before harvest for IB assay. Tubulin served as loading control. **(D)** IB analyses of HEK293T cells transfected with plasmids encoding HA-p62 and ASB6 mutants. Tubulin served as a loading control. **(E,F)** HEK293T cells were transfected with plasmids encoding HA-p62 and EV or FLAG-ASB6. Forty-eight hours after transfection, cells were treated with 50 μg/ml CHX and harvested at the indicated time for IB analysis. Tubulin served as loading control. Relative HA-p62 protein levels were quantified and normalized to Tubulin with ImageJ software. **(G)** IB analysis of HEK293T cells transfected with a plasmid encoding FLAG-ASB6. Tubulin served as a loading control. **(H)** HEK293T cells transfected with the indicated plasmids were treated with 10 μM MG132 for 10 h before harvest for Ni-NTA beads pull down and IB analysis.

Next, a series of experiments were carried out to determine whether ASB6 is a key p62 regulator. Transient co-transfection of multiple shRNA against ASB6 caused dramatic upregulation of ectopically expressed HA-p62 in 293T cells (Figure 3A). Consistently, stable depletion of ASB6 also significantly increased endogenous p62 protein in HeLa cells (Figure 3B), with ASB6 knockdown efficiency confirmed by RT-PCR. On the other hand, co-expression of FLAG-ASB6 caused degradation of ectopically expressed HA-p62 in 293T cells, which could be blocked by proteasome inhibitor MG132 in a dose-dependent manner (Figure 3C). Moreover, deletion of either SOCS-box or the smaller Cul5-box impaired the capacity of ASB6 to degrade p62 (Figure 3D), which may due to the damaged potential to form functional CRL E3 complex with CUL5 (Figure 2D). We also transfected various amounts of FLAG-ASB6 constructs in 293T cells and examined the expression changes of endogenous p62. As shown in Figure 3G, the decrease of endogenous p62 is inversely correlated with the amount of overexpressed ASB6. We further performed CHX chase experiment to determine if the observed reduced p62 expression is caused by p62 protein stability change. As indicated in Figures 3E,F, p62 is a very stable protein without overexpression of ASB6, and its half-life was significantly shortened in the presence of ASB6. So these results suggested that ASB6 negatively regulates p62 protein stability. We subsequently confirmed that overexpression of CUL5 and ASB6 could promote ubiquitination of endogenous p62 (Figure 3H). Therefore, our data suggest that the CUL5–ASB6 complex is a functional E3 ligase promotes ubiquitination and degradation of p62.

**FIGURE 4:**
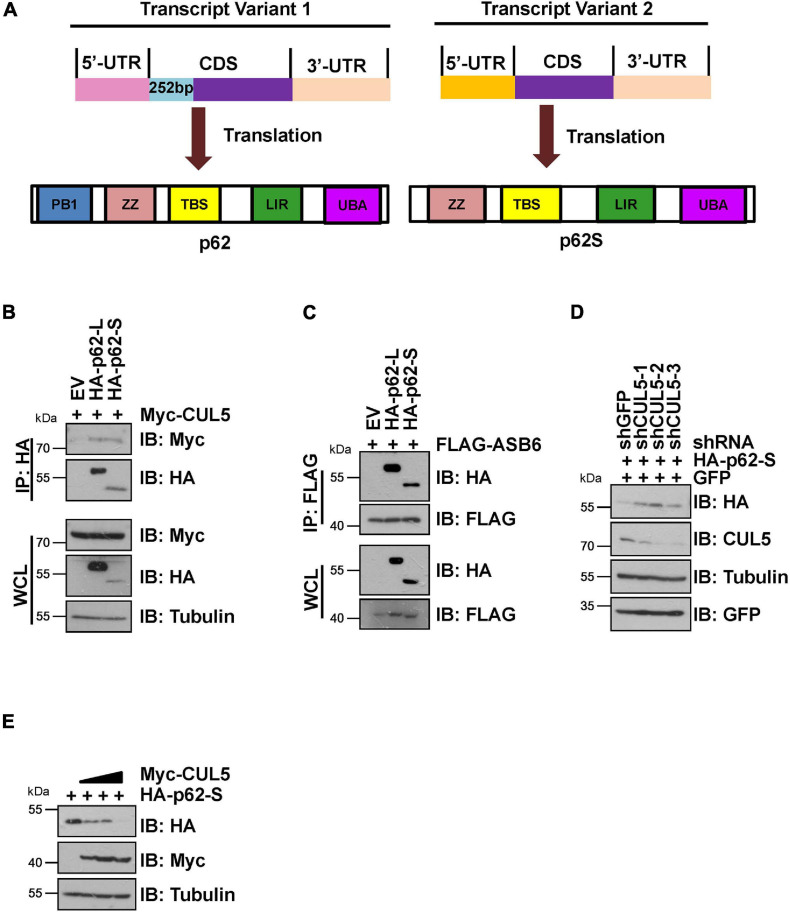
CUL5–ASB6 complex interacts with and regulates short p62 isoform. **(A)** Schematic representation of the domain composition of p62 and p62S. **(B,C)** HEK293T cells transfected with the indicated plasmids were treated with 10 μM MG132 for 10 h before harvest for IP and IB analysis. **(D)** HEK293T cells were co-transfected with plasmids encoding HA-p62S, shRNAs targeting CUL5, and GFP (as an internal transfection control), and were harvested for IB analysis 48 h later. Tubulin served as a loading control. **(E)** IB analysis of HEK293T cells transfected with HA-p62S plasmid and increasing amount of Myc-CUL5 construct. Tubulin served as a loading control.

It has been reported that p62 has two protein isoforms that are generated by three mRNA variants due to alternative splicing (Wang et al., 2014). Different from the commonly detected 440 amino acid isoform, the shorter p62 isoform (p62S) lacks the N-terminal PB1 domain and contains 356 aa in length (Figure 4A). Therefore, we examined whether p62S is also subject to CUL5–ASB6 mediated regulation. As indicated, p62S also interacted with CUL5 and ASB6, in a manner very similar to the longer p62 isoform (Figures 4B,C). Co-transfection of CUL5 shRNAs can also dramatically upregulate HA-p62S expression (Figure 4D), while overexpression of CUL5 reduced HA-p62S protein level (Figure 4E). These results suggested that the CUL5–ASB6 E3 ligase regulates not only the classic p62 protein but also the shorter p62 isoform.

### ASB6-Mediated p62 Degradation Is Independent of Autophagy

**FIGURE 5:**
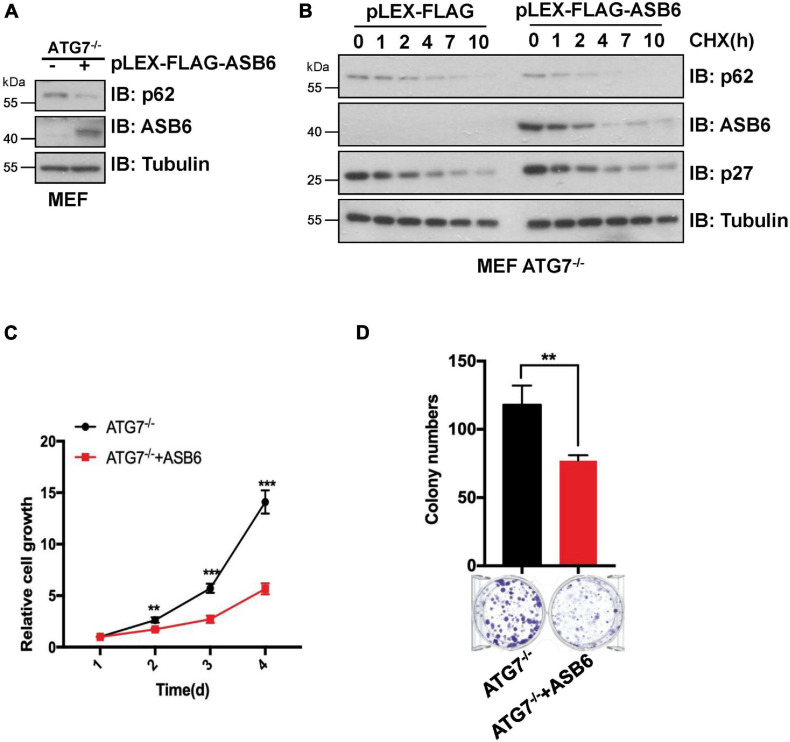
The degradation of p62 by ASB6 is independent of autophagy. **(A)** IB analysis of p62 levels in ATG7^–/–^ MEF cells stably expressing ectopic ASB6. Tubulin was used as a loading control. **(B)** ATG7^–/–^ MEF cells stably expressing ectopic ASB6 were treated with 50 μg/ml CHX and harvested at indicated time for IB analysis. Tubulin served as loading control. **(C)** ATG7^–/–^ MEF cells stably expressing ectopic ASB6 were counted and seeded into 96-well plates (500 cells per well) to perform CCK8 experiment at the indicated time. Data were shown as mean ± SEM (*n* = 3). Statistical analyses were performed using Student’s *t*-test. **P < 0.01, ***P < 0.001. **(D)** ATG7^–/–^ MEF cells stably overexpressing ASB6 were counted and seeded into six-well plates (500 cells per well). The number of colonies were measured and analyzed after 10 days. Data represent the mean ± SEM. **P < 0.01, by Student’s *t*-test.

p62 is the most famous autophagy receptor protein and itself also gets degraded in the autophagosomes together with the cargo protein. So, decrease of p62 protein is regarded as an indicator of autophagy process. To dissect whether CUL5–ASB6-mediated p62 degradation depends on autophagy process, we performed multiple experiments with ATG7 knockout (ATG7^–/–^) MEF cells, in which autophagy pathway is defective due to loss of ATG7, a ubiquitin E1-like activating enzyme essential for the assembly and function of ubiquitin-like conjugation systems during autophagy (Nakatogawa et al., 2009). We found that overexpression of ASB6 could downregulate p62 protein levels in ATG7^–/–^ MEF cells and shorten p62 half-life in the CHX chase experiment (Figures 5A,B). Meanwhile, ASB6 overexpression also inhibited the colony formation and cell proliferation of ATG7^–/–^ MEF cells (Figures 5C,D). These results suggest that the regulation of p62 by the CUL5–ASB6 complex does not depend on the occurrence of autophagy process. Therefore, the CUL5–ASB6 complex may regulate cell proliferation *via* p62 independent of autophagy.

### ASB6 Modulates Hepatocellular Carcinoma Cell Proliferation and Autophagy *via* p62

**FIGURE 6:**
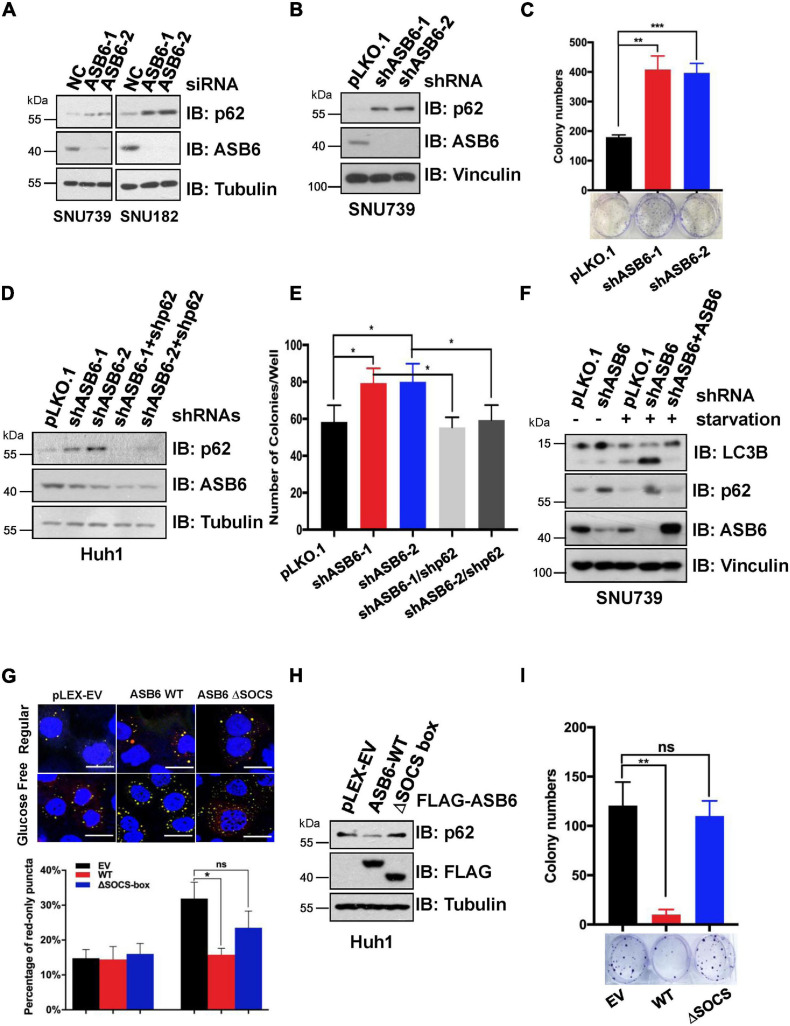
ASB6 functions in HCC cells by regulating p62. **(A)** IB analysis of HCC cell lines SNU739 and SNU182 that were transfected with ASB6 siRNAs. Tubulin was used as loading control. **(B)** SNU739 cells infected with indicated shRNAs were harvested for IB analysis. Vinculin served as a loading control. **(C)** The cells from **(B)** were counted and seeded into six-well plates (500 cells per well) to perform colony formation assay. The number of colonies were counted and analyzed. Data represent the mean ± SEM. **P < 0.01, ***P < 0.001, by Student’s *t*-test. **(D)** Huh1 cells were treated with lentiviral shRNAs against ASB6 and p62. The resulting cells were harvested and analyzed by IB with indicated antibodies. Tubulin served as loading control. **(E)** The cells from **(D)** were counted and transferred to six-well plates (500 cells per well) to perform colony formation assay. The number of colonies was measured and analyzed after 10 days. Data represent the mean ± SEM. *P < 0.05, by Student’s *t*-test. **(F)** IB analysis of SNU739 cells pre-treated with ASB6 stably knockdown and ASB6 re-expression (a mutant resistant to shRNA treatment). Resulting cells were treated with glucose deprivation for 16 h to induce autophagy. Vinculin was used as loading control. **(G)** Huh1 cells stably expressing mCherry-GFP-LC3 were treated with viral vectors encoding ectopic FLAG-ASB6 WT or ΔSOCS-box mutant. Glucose deprivation was performed for 16 h to induce autophagy. GFP and mCherry puncta were measured and analyzed under confocal microscope, and the average percentage of cells containing red-only puncta, which represents the matured antophagosome, was determined and blotted. Scale bars, 20 μm. Data represent the mean ± SEM. ns, non-significant; *P < 0.05, by Student’s *t*-test. **(H)** IB analysis of Huh1 cells generated in **(G)**. Tubulin was used as loading control. **(I)** The cells from **(G)** were counted and transferred to six-well plates (500 cells per well) to perform colony formation assay. The number of colonies was measured and analyzed after 10 days. Data represent the mean ± SEM. ns, non-significant; **P < 0.01, by Student’s *t*-test.

Since p62 has been reported to play an important role in the occurrence and development of liver cancer, we next investigated whether ASB6 has a function in liver cancer cells by regulating p62. We first depleted ASB6 in HCC cell lines SNU739 and SNU182 with siRNAs and shRNAs that target ASB6 (Figures 6A,B) and observed significant upregulation of p62 protein. Importantly, depletion of ASB6 with shRNA greatly increased the colony formation of SNU739 cells (Figure 6C). Moreover, further depletion of p62 completely reversed the promoted colony formation caused by ASB6 knockdown in Huh1 cells (Figures 6D,E). Therefore, the increased cell proliferation by ASB6 knockdown is largely due to p62 upregulation.

As p62 is a well-characterized factor in autophagy, we further investigated whether ASB6-mediated p62 degradation would affect the autophagy process. When treated with glucose starvation condition, more advanced autophagy occurred in ASB6-depleted SNU739 cells as evidenced by the much increased shorter LC3-II isoform compared to pLKO.1 vector-treated control cells, and further overexpression of ASB6 could indeed inhibit the LC3-II protein level (Figure 6F). This result indicated that ASB6 may be an inhibitory factor for autophagy. To further prove this point, we constructed a Huh1 cell line stable expressing the autophagy fluorescent reporter protein mCherry-GFP-LC3, and then ectopically expressed FLAG tagged ASB6 wild type and ΔSOCS-box ASB6 truncate in it. The expression of endogenous p62 was examined and autophagy was induced by removing glucose from culture medium in these resulting cells. As indicated, expression of ASB6 wild type but not ΔSOCS-box ASB6 truncate reduced p62 expression (Figure 6H). More importantly, the number of mature autophagosomes (marked by red-only puncta, since GFP protein is denatured in acidic condition) significantly reduced in wild-type ASB6 expressed cells (Figure 6G). Consistently, expression of ASB6 wild type, but not ΔSOCS-box ASB6 truncate, also strongly reduced the colony formation of the resulted Huh1 cells (Figure 6I). Therefore, these data suggested that ASB6 has an inhibitory role in autophagy and cell proliferation possibly *via* governing p62 abundance in HCC cells.

## Discussion

**FIGURE 7:**
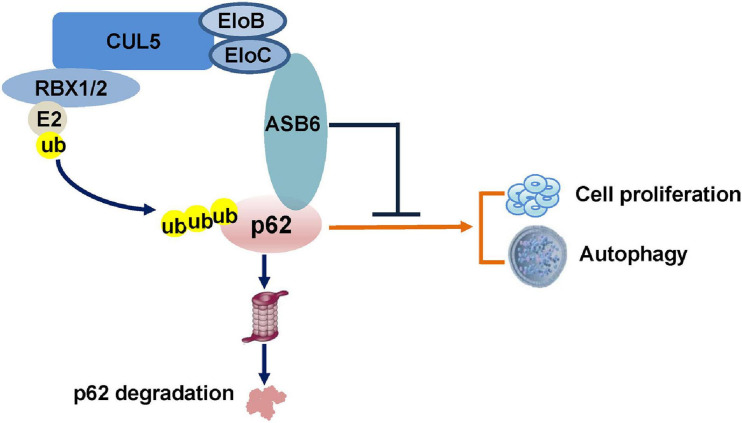
Schematic model of the CRL5–ASB6 E3 complex mediated p62 degradation.

In the current study, we identified a new functional ubiquitin E3 ligase complex, CRL5–ASB6 complex, as a major regulator governing p62/SQSTM1 abundance (Figure 7). As the largest E3 ligase family, Cullin-Ring E3 ligase complexes are composed of hundreds of members differing in various Cullin proteins, and more importantly, the substrate recognition proteins (Nguyen et al., 2017). Initially designated as VACM-1, CUL5 is a relatively late identified member for the Cullin family (Burnatowska-Hledin et al., 1995). The following studies revealed that CUL5 forms a series of CRL5 E3 ligase complex together with RING protein RBX1/2, adaptor proteins EloB/C, and, most importantly, the substrate recognition subunit SOCS-box proteins. With more and more studies published, various roles of CRL5 E3 ligase in viral infection, signaling transduction, and carcinomagenesis have been revealed (Zhao and Sun, 2013). Although CUL5 protein is found downregulated in multiple cancers, the exact function of each CRL5 E3 ligase complex in oncogenesis is very diverse since CRL5 E3 ligases could promote the degradation of both oncoproteins and tumor suppressors. For example, we have found that the CRL5–SOCS3 complex degrades ITGB1 to inhibit small cell lung cancer cell migration and metastasis, and the CRL5–SOCS6 complex governs Sin1 level and limits mTORC2 function (Cui et al., 2019; Zhao et al., 2019). Moreover, other groups found that the CRL5–ASB13 complex degrades SNAI2 and the CRL5–SPSB3 complex degrades SNAIL to inhibit cancer metastasis (Liu et al., 2018; Fan et al., 2020). These evidences suggested that certain CRL5 E3 complexes could exert tumor suppressor function in cancer cells. On the other hand, CRL5–WSB1 has been shown to promote tumor metastasis and promote cell cycle *via* degrading tumor suppressors VHL, RhoGDI2, ATM, etc. (Cao et al., 2015; Kim et al., 2015, 2017). Therefore, the role of CRL5 E3 complexes in cancer regulation is very complicated and possibly context dependent. Interestingly, CUL5 has also been implicated in regulating autophagy by promoting the turnover of mTORC1 inhibitor DEPTOR, a process that could be blocked by AMBRA1 (Antonioli et al., 2014). Notably, the reported CUL5–DEPTOR–mTORC1 regulation of autophagy is rather indirect and related to the onset stage of autophagy, since it mainly goes *via* phosphorylation and inhibition of ULK1 (Antonioli et al., 2014). Therefore, our study established a new regulatory role of CUL5 in autophagy *via* governing p62.

ASB6 contains six ankyrin repeats at its N-terminal and a SOCS-box at its C-terminal, which enable it to be integrated as part of the CRL5 E3 complex *via* EloB/C. ASB6 was first identified *via* yeast two hybrid as an interaction protein of APS (also known as SH2B2), an adaptor protein that is involved in the activation of multiple tyrosine kinases (Wilcox et al., 2004). The interaction between APS and ASB6 was further validated by immunoprecipitation and immunofluorescence methods, and ASB6 was shown to possibly recruit EloB/C and other binding partners to the plasma membrane to cause the degradation of APS and ASB6 itself upon activation of insulin receptor. Later, ASB6 was found upregulated in oral squamous cell carcinoma, which is a possible consequence of exposure to Areca nut extracts and is co-related with poor survival (Hung et al., 2009). Another recent following study from the same group suggested that accumulation of ASB6 may impede ER stress and cause gain of stemness and metastasis feature of oral squamous cell carcinoma, although the underlying molecular mechanism remains elusive (Hung et al., 2019). Therefore, the detailed molecular function of ASB6, especially in pathological conditions, is largely unknown. In the current study, we characterized ASB6 as a substrate recognition subunit of CRL5 E3 ligase that governs p62 abundance. Notably, depletion of ASB6 promotes colony formation of HCC cell line Huh1, which could be reversed by further knockdown of p62, indicating that the function of ASB6 in inhibiting HCC cell proliferation is largely through p62. Consistent with the necessary role of p62 in promoting autophagy, overexpression of ASB6 (but not the ΔSOCS-box ASB6 truncate) degrades endogenous p62 and suppresses proceeding of autophagy and colony formation in Huh1 cells. So, it is possible that the function of ASB6 in control cell proliferation–autophagy homeostasis is dependent on p62 status. Nevertheless, a more mechanistic study is required to fully address the biological meaning of the CRL5–ASB6–p62 regulation in physiological and pathological conditions.

Taken together, we have identified the CRL5–ASB6 complex as a functional ubiquitin E3 ligase that promotes the ubiquitination and degradation of p62, through which it regulates cell proliferation and autophagy. Our finding may shed new light on understanding the complex regulation of p62 function and cell proliferation–autophagy homeostasis.

## Data Availability Statement

The raw data supporting the conclusions of this article will be made available by the authors, without undue reservation.

## Author Contributions

LG conducted the experiments with help from KW, MW, and HL. DG and ML supervised the project with intellectual input from RH. DG, LG, and ML wrote the manuscript with input from the other authors. All authors contributed to the article and approved the submitted version.

## Conflict of Interest

The authors declare that the research was conducted in the absence of any commercial or financial relationships that could be construed as a potential conflict of interest.

## References

[B1] Aigelsreiter A. Neumann J. Pichler M. Halasz J. Zatloukal K. Berghold A. (2017). Hepatocellular carcinomas with intracellular hyaline bodies have a poor prognosis. *Liver Int.* 37 600–610. 10.1111/liv.13325 27885796

[B2] Antonioli M. Albiero F. Nazio F. Vescovo T. Perdomo A. B. Corazzari M. (2014). AMBRA1 interplay with cullin E3 ubiquitin ligases regulates autophagy dynamics. *Dev. Cell* 31 734–746. 10.1016/j.devcel.2014.11.013 25499913

[B3] Burnatowska-Hledin M. A. Spielman W. S. Smith W. L. Shi P. Meyer J. M. Dewitt D. L. (1995). Expression cloning of an AVP-activated, calcium-mobilizing receptor from rabbit kidney medulla. *Am. J. Physiol.* 268 F1198–F1210. 7611460 10.1152/ajprenal.1995.268.6.F1198

[B4] Cao J. Wang Y. Dong R. Lin G. Zhang N. Wang J. (2015). Hypoxia-induced WSB1 promotes the metastatic potential of osteosarcoma cells. *Cancer Res.* 75 4839–4851. 10.1158/0008-5472.can-15-0711 26424695

[B5] Cui B. Gong L. Chen M. Zhang Y. Yuan H. Qin J. (2019). CUL5-SOCS6 complex regulates mTORC2 function by targeting sin1 for degradation. *Cell Discov.* 5:52. 31798957 10.1038/s41421-019-0118-6 PMC6868212

[B6] Du P. Wang L. Sliz P. Gregory R. I. (2015). A biogenesis step upstream of microprocessor controls mir-17 approximately 92 expression. *Cell* 162 885–899. 10.1016/j.cell.2015.07.008 26255770 PMC4537828

[B7] Fan H. Wang X. Li W. Shen M. Wei Y. Zheng H. (2020). ASB13 inhibits breast cancer metastasis through promoting SNAI2 degradation and relieving its transcriptional repression of YAP. *Genes Dev.* 34 1359–1372. 10.1101/gad.339796.120 32943576 PMC7528707

[B8] Heath R. J. Goel G. Baxt L. A. Rush J. S. Mohanan V. Paulus G. L. C. (2016). RNF166 determines recruitment of adaptor proteins during antibacterial autophagy. *Cell Rep.* 17 2183–2194. 10.1016/j.celrep.2016.11.005 27880896 PMC5192565

[B9] Hershko A. Ciechanover A. (1998). The ubiquitin system. *Annu. Rev. Biochem.* 67 425–479. 9759494 10.1146/annurev.biochem.67.1.425

[B10] Huang J. Diaz-Meco M. T. Moscat J. (2018). The macroenviromental control of cancer metabolism by p62. *Cell Cycle* 17 2110–2121. 10.1080/15384101.2018.1520566 30198373 PMC6226228

[B11] Huang X. Wang X. N. Yuan X. D. Wu W. Y. Lobie P. E. Wu Z. (2019). XIAP facilitates breast and colon carcinoma growth via promotion of p62 depletion through ubiquitination-dependent proteasomal degradation. *Oncogene* 38 1448–1460. 10.1038/s41388-018-0513-8 30275562

[B12] Hung K. F. Lai K. C. Liu T. Y. Liu C. J. Lee T. C. Lo J. F. (2009). Asb6 upregulation by *Areca* nut extracts is associated with betel quid-induced oral carcinogenesis. *Oral Oncol* 45 543–548. 10.1016/j.oraloncology.2008.10.004 19251471

[B13] Hung K. F. Liao P. C. Chen C. K. Chiu Y. T. Cheng D. H. Kawasumi M. (2019). ASB6 promotes the stemness properties and sustains metastatic potential of oral squamous cell carcinoma cells by attenuating ER stress. *Int. J. Biol. Sci.* 15 1080–1090. 10.7150/ijbs.31484 31182927 PMC6535794

[B14] Inami Y. Waguri S. Sakamoto A. Kouno T. Nakada K. Hino O. (2011). Persistent activation of Nrf2 through p62 in hepatocellular carcinoma cells. *J. Cell Biol.* 193 275–284. 10.1083/jcb.201102031 21482715 PMC3080263

[B15] Inoue D. Suzuki T. Mitsuishi Y. Miki Y. Suzuki S. Sugawara S. (2012). Accumulation of p62/SQSTM1 is associated with poor prognosis in patients with lung adenocarcinoma. *Cancer Sci.* 103 760–766. 10.1111/j.1349-7006.2012.02216.x 22320446 PMC7659245

[B16] Jadhav T. Geetha T. Jiang J. Wooten M. W. (2008). Identification of a consensus site for TRAF6/p62 polyubiquitination. *Biochem. Biophys. Res. Commun.* 371 521–524. 10.1016/j.bbrc.2008.04.138 18457658 PMC2474794

[B17] Jain A. Lamark T. Sjottem E. Larsen K. B. Awuh J. A. Overvatn A. (2010). p62/SQSTM1 is a target gene for transcription factor NRF2 and creates a positive feedback loop by inducing antioxidant response element-driven gene transcription. *J. Biol. Chem.* 285 22576–22591. 10.1074/jbc.m110.118976 20452972 PMC2903417

[B18] Jongsma M. L. Berlin I. Wijdeven R. H. Janssen L. Janssen G. M. Garstka M. A. (2016). An ER-associated pathway defines endosomal architecture for controlled cargo transport. *Cell* 166 152–166. 27368102 10.1016/j.cell.2016.05.078 PMC4930482

[B19] Kim J. J. Lee S. B. Jang J. Yi S. Y. Kim S. H. Han S. A. (2015). WSB1 promotes tumor metastasis by inducing pVHL degradation. *Genes Dev.* 29 2244–2257. 10.1101/gad.268128.115 26545811 PMC4647558

[B20] Kim J. J. Lee S. B. Yi S. Y. Han S. A. Kim S. H. Lee J. M. (2017). WSB1 overcomes oncogene-induced senescence by targeting ATM for degradation. *Cell Res.* 27 274–293. 10.1038/cr.2016.148 27958289 PMC5339850

[B21] Kitamura H. Torigoe T. Asanuma H. Hisasue S. I. Suzuki K. Tsukamoto T. (2006). Cytosolic overexpression of p62 sequestosome 1 in neoplastic prostate tissue. *Histopathology* 48 157–161. 10.1111/j.1365-2559.2005.02313.x 16405664

[B22] Komatsu M. (2011). Potential role of p62 in tumor development. *Autophagy* 7 1088–1090. 10.4161/auto.7.9.16474 21617386

[B23] Kraft L. J. Dowler J. Manral P. Kenworthy A. K. (2016). Size, organization, and dynamics of soluble SQSTM1 and LC3-SQSTM1 complexes in living cells. *Autophagy* 12 1660–1674. 10.1080/15548627.2016.1199299 27442348 PMC5082789

[B24] Layfield R. Hocking L. J. (2004). SQSTM1 and Paget’s disease of bone. *Calcif. Tissue Int.* 75 347–357. 10.1007/s00223-004-0041-0 15365659

[B25] Lee Y. Chou T. F. Pittman S. K. Keith A. L. Razani B. Weihl C. C. (2017). Keap1/Cullin3 modulates p62/SQSTM1 activity via UBA domain ubiquitination. *Cell Rep.* 20:1994. 10.1016/j.celrep.2017.08.019 28834760

[B26] Li L. J. Shen C. Nakamura E. Ando K. Signoretti S. Beroukhim R. (2013). SQSTM1 is a pathogenic target of 5q copy number gains in kidney cancer. *Cancer Cell* 24 738–750. 10.1016/j.ccr.2013.10.025 24332042 PMC3910168

[B27] Lim J. Lachenmayer M. L. Wu S. Liu W. Kundu M. Wang R. (2015). Proteotoxic stress induces phosphorylation of p62/SQSTM1 by ULK1 to regulate selective autophagic clearance of protein aggregates. *PLoS Genet.* 11:e1004987. 10.1371/journal.pgen.1004987 25723488 PMC4344198

[B28] Lin Q. Dai Q. Meng H. Sun A. Wei J. Peng K. (2017). The HECT E3 ubiquitin ligase NEDD4 interacts with and ubiquitylates SQSTM1 for inclusion body autophagy. *J. Cell. Sci.* 130 3839–3850. 29021346 10.1242/jcs.207068

[B29] Linares J. F. Duran A. Yajima T. Pasparakis M. Moscat J. Diaz-Meco M. T. (2013). K63 polyubiquitination and activation of mTOR by the p62-TRAF6 complex in nutrient-activated cells. *Mol. Cell* 51 283–296. 10.1016/j.molcel.2013.06.020 23911927 PMC3971544

[B30] Liu Y. Kern J. T. Walker J. R. Johnson J. A. Schultz P. G. Luesch H. (2007). A genomic screen for activators of the antioxidant response element. *Proc. Natl. Acad. Sci. U.S.A.* 104 5205–5210. 10.1073/pnas.0700898104 17360324 PMC1829287

[B31] Liu Y. Zhou H. Zhu R. Ding F. Li Y. Cao X. (2018). SPSB3 targets SNAIL for degradation in GSK-3beta phosphorylation-dependent manner and regulates metastasis. *Oncogene* 37 768–776. 10.1038/onc.2017.370 29059170

[B32] Mathew R. Karp C. M. Beaudoin B. Vuong N. Chen G. Chen H. Y. (2009). Autophagy suppresses tumorigenesis through elimination of p62. *Cell* 137 1062–1075. 10.1016/j.cell.2009.03.048 19524509 PMC2802318

[B33] Matsumoto G. Wada K. Okuno M. Kurosawa M. Nukina N. (2011). Serine 403 phosphorylation of p62/SQSTM1 regulates selective autophagic clearance of ubiquitinated proteins. *Mol. Cell* 44 279–289. 10.1016/j.molcel.2011.07.039 22017874

[B34] McManus S. Roux S. (2012). The adaptor protein p62/SQSTM1 in osteoclast signaling pathways. *J. Mol. Signal.* 7:1. 10.1186/1750-2187-7-1 22216904 PMC3309942

[B35] Morreale F. E. Walden H. (2016). Types of ubiquitin ligases. *Cell* 165:248.e1. 27015313 10.1016/j.cell.2016.03.003

[B36] Moscat J. Diaz-Meco M. T. (2012). p62: a versatile multitasker takes on cancer. *Trends Biochem. Sci.* 37 230–236. 10.1016/j.tibs.2012.02.008 22424619 PMC3531712

[B37] Nakatogawa H. Suzuki K. Kamada Y. Ohsumi Y. (2009). Dynamics and diversity in autophagy mechanisms: lessons from yeast. *Nat. Rev. Mol. Cell Biol.* 10 458–467. 10.1038/nrm2708 19491929

[B38] Nguyen H. C. Wang W. Xiong Y. (2017). Cullin-RING E3 ubiquitin ligases: bridges to destruction. *Subcell. Biochem.* 83 323–347. 10.1007/978-3-319-46503-6_12 28271482 PMC7205596

[B39] Pan J. A. Sun Y. Jiang Y. P. Bott A. J. Jaber N. Dou Z. (2016). TRIM21 ubiquitylates SQSTM1/p62 and suppresses protein sequestration to regulate redox homeostasis. *Mol. Cell* 62 149–151. 10.1016/j.molcel.2016.03.015 27058791

[B40] Park S. Choi S. G. Yoo S. M. Son J. H. Jung Y. K. (2014). Choline dehydrogenase interacts with SQSTM1/p62 to recruit LC3 and stimulate mitophagy. *Autophagy* 10 1906–1920. 10.4161/auto.32177 25483962 PMC4502719

[B41] Pilli M. Arko-Mensah J. Ponpuak M. Roberts E. Master S. Mandell M. A. (2012). TBK-1 promotes autophagy-mediated antimicrobial defense by controlling autophagosome maturation. *Immunity* 37 223–234. 10.1016/j.immuni.2012.04.015 22921120 PMC3428731

[B42] Ren F. Shu G. Liu G. Liu D. Zhou J. Yuan L. (2014). Knockdown of p62/sequestosome 1 attenuates autophagy and inhibits colorectal cancer cell growth. *Mol. Cell. Biochem.* 385 95–102. 10.1007/s11010-013-1818-0 24065390

[B43] Ro S. H. Semple I. A. Park H. Park H. Park H. W. Kim M. (2014). Sestrin2 promotes Unc-51-like kinase 1 mediated phosphorylation of p62/sequestosome-1. *FEBS J.* 281 3816–3827. 10.1111/febs.12905 25040165 PMC4156532

[B44] Rolland P. Madjd Z. Durrant L. Ellis I. O. Layfield R. Spendlove I. (2007). The ubiquitin-binding protein p62 is expressed in breast cancers showing features of aggressive disease. *Endocr. Relat. Cancer* 14 73–80. 10.1677/erc.1.01312 17395976

[B45] Sanchez-Martin P. Saito T. Komatsu M. (2019). p62/SQSTM1: ‘Jack of all trades’ in health and cancer. *FEBS J.* 286 8–23. 10.1111/febs.14712 30499183 PMC7379270

[B46] Sanz L. Sanchez P. Lallena M. J. Diaz-Meco M. T. Moscat J. (1999). The interaction of p62 with RIP links the atypical PKCs to NF-kappaB activation. *EMBO J.* 18 3044–3053. 10.1093/emboj/18.11.3044 10356400 PMC1171386

[B47] Schulman B. A. Harper J. W. (2009). Ubiquitin-like protein activation by E1 enzymes: the apex for downstream signalling pathways. *Nat. Rev. Mol. Cell Biol.* 10 319–331. 10.1038/nrm2673 19352404 PMC2712597

[B48] Song P. Li S. Wu H. Gao R. Rao G. Wang D. (2016). Parkin promotes proteasomal degradation of p62: implication of selective vulnerability of neuronal cells in the pathogenesis of Parkinson’s disease. *Protein Cell* 7 114–129. 10.1007/s13238-015-0230-9 26746706 PMC4742389

[B49] Soucy T. A. Smith P. G. Milhollen M. A. Berger A. J. Gavin J. M. Adhikari S. (2009). An inhibitor of NEDD8-activating enzyme as a new approach to treat cancer. *Nature* 458 732–736. 19360080 10.1038/nature07884

[B50] Umemura A. He F. Taniguchi K. Nakagawa H. Yamachika S. Font-Burgada J. (2016). p62, upregulated during preneoplasia, induces hepatocellular carcinogenesis by maintaining survival of stressed hcc-initiating cells. *Cancer Cell* 29 935–948. 10.1016/j.ccell.2016.04.006 27211490 PMC4907799

[B51] Wang L. Cano M. Handa J. T. (2014). p62 provides dual cytoprotection against oxidative stress in the retinal pigment epithelium. *Biochim. Biophys. Acta.* 1843 1248–1258. 10.1016/j.bbamcr.2014.03.016 24667411 PMC4019388

[B52] Wenzel D. M. Stoll K. E. Klevit R. E. (2011). E2s: structurally economical and functionally replete. *Biochem. J.* 433 31–42. 10.1042/bj20100985 21158740 PMC3118098

[B53] Wilcox A. Katsanakis K. D. Bheda F. Pillay T. S. (2004). Asb6, an adipocyte-specific ankyrin and SOCS box protein, interacts with APS to enable recruitment of elongins B and C to the insulin receptor signaling complex. *J. Biol. Chem.* 279 38881–38888. 10.1074/jbc.m406101200 15231829

[B54] Yan X. Y. Zhong X. R. Yu S. H. Zhang L. C. Liu Y. N. Zhang Y. (2019). p62 aggregates mediated caspase 8 activation is responsible for progression of ovarian cancer. *J. Cell. Mol. Med.* 23 4030–4042. 10.1111/jcmm.14288 30941888 PMC6533521

[B55] Zhao G. Gong L. Su D. Jin Y. Guo C. Yue M. (2019). Cullin5 deficiency promotes small-cell lung cancer metastasis by stabilizing integrin beta1. *J. Clin. Invest.* 129 972–987. 10.1172/jci122779 30688657 PMC6391098

[B56] Zhao Y. Sun Y. (2013). Cullin-RING Ligases as attractive anti-cancer targets. *Curr. Pharm. Des.* 19 3215–3225. 10.2174/13816128113199990300 23151137 PMC4034125

